# Molecular detection of *Wolbachia* and *Bartonella* as part of the microbiome of phlebotomine sand flies from Chiapas, Mexico

**DOI:** 10.1007/s00436-023-07829-z

**Published:** 2023-04-14

**Authors:** Yokomi N. Lozano-Sardaneta, Carlos F. Marina, Jorge A. Torres-Monzón, Víctor Sánchez-Cordero, Ingeborg Becker

**Affiliations:** 1 Centro de Medicina Tropical, Unidad de Medicina Experimental, Facultad de Medicina de la Universidad Nacional Autónoma de México, Hospital General de México, Dr. Balmis 148, Col. Doctores, 06726 Mexico City, México; 2 Centro Regional de Investigación en Salud Pública, Instituto Nacional de Salud Pública (CRISP-INSP), Tapachula, Chiapas México; 3 Instituto de Biología, Universidad Nacional Autónoma de México, Ciudad de Mexico, 04510 México

**Keywords:** *Wolbachia*, *Bartonella*, Vector, Food sources, *Lutzomyia*

## Abstract

Phlebotomine sand flies are dipterans of relevance due to their role as vectors of several pathogens worldwide. Bacteria in the gut of sand flies possibly affect their vectorial capacity and competence to transmit parasites. A retrospective study was performed in sand fly specimens that had previously been collected in four localities of the state of Chiapas during the period 2009–2011 to detect *Wolbachia* and *Bartonella* and their possible coinfection with *Leishmania*. For the molecular detection of bacteria, we used primers and conditions that had previously been reported. A total of 531 sand fly specimens of 10 species were analyzed. Four *Wolbachia* strains were detected in five sand fly species, showing a prevalence of 8.6%. All the *Wolbachia* strains had previously been reported in other taxa. In one sand fly species, we also detected a new lineage of *Bartonella* evidenced by a phylogenetic analysis. No sand fly specimens showed coinfections of these bacteria and *Leishmania*. The bacteria found in the phlebotomine sand flies are possibly transmitted by plant-mediated horizontal transmission and during blood meal feeding.

## Introduction

Phlebotomine sand flies (Diptera: Psychodidae, Phlebotominae) are vectors of several pathogens such as *Leishmania*, *Bartonella*, and some arboviruses that affect human health worldwide (Akhoundi et al. 2016). In recent years, comprehensive studies have revealed basic aspects of the life cycle of this vector, helping to establish efficient prevention and control strategies to avoid the transmission of these pathogens.

The analysis of the diversity of bacteria in the gut of sand fly species provides insights into their vectorial capacity and competence to transmit parasites (Sallum et al. 2019; Vivero et al. 2019). For instance, the detection of the *α*-proteobacteria *Wolbachia* is relevant, since this bacterium spreads quickly and manipulates the reproductive success of its insect host, in order to guarantee its own propagation, causing reproductive alterations such as cytoplasmic incompatibility (CI), induction of parthenogenesis, and feminization or death of males (Werren 1997; Pimentel et al. 2020). These effects represent a possible strategy for the biological control of sand flies. The presence of *Wolbachia* in some mosquitoes of the genus *Culex* and *Aedes* has been reported to confer protection against nematodes and virus transmission, such as in dengue, preventing its replication (Karimian et al. 2018; Pimentel et al. 2020).

For that reason, it has been proposed that the association between *Wolbachia* and sand flies probably interferes with the establishment of *Leishmania* species. In the American continent, only 11 sand fly species have been associated with *Wolbachia* strains in Brazil, Colombia, Mexico, and Panama, although the role of these associations remains unclear (Ono et al. 2001; Azpurua et al. 2010; Mikery-Pacheco et al. 2012; Monteiro et al. 2016; Kelly et al. 2017; Vivero et al. 2017; Lozano-Sardaneta et al. 2021a, b; Lozano-Sardaneta et al. 2022). Recent studies propose that *Wolbachia* in sand flies might induce cytoplasmic incompatibility that reduces the genetic variability, causing speciation (Kassem et al. 2003; Azpurua et al. 2010). In Mexico, only *Lutzomyia cruciata* and *Psathyromyia shannoni* have been reported to be infected with the strain *Wolbachia* wWhi in the states of Chiapas, Veracruz, and Tabasco (Mikery-Pacheco et al. 2012; Lozano-Sardaneta et al. 2021a, b; Lozano-Sardaneta et al. 2022). This is relevant, since both sand flies are considered to be vectors of *Leishmania mexicana* and *Leishmania infantum* in Mexico (Pech-May et al. 2010; Lozano-Sardaneta et al. 2020).

Another bacterium transmitted by a sand fly is *Bartonella*, a hemotropic bacterium that causes chronic intraerythrocytic infections in their hosts (Chomel et al. 2009). The only confirmed species causing bartonellosis transmitted by sand flies are *Bartonella bacilliformis* and *Bartonella grahamii*, which are endemic species in the Andean valleys (Lozano-Sardaneta et al. 2019). Uncultured *Bartonella* spp. have recently been recorded in association with *Lu. cruciata* and *Pa. shannoni* in the states of Veracruz and Tamaulipas, Mexico, although it remains to be established whether the sand flies are vectors of these bacteria and if these *Bartonella* species cause emerging diseases (Lozano-Sardaneta et al. 2019; Lozano-Sardaneta et al. 2021a).

Chiapas is the Mexican state that harbors the highest number of sand fly species (36 of 52) and is also considered an endemic area of leishmaniasis (Ibáñez-Bernal et al. 2015). Therefore, the aim of this study was to conduct a retrospective study focused on the molecular detection of the bacteria of the genus *Wolbachia* and *Bartonella* in sand flies and their possible coinfection with *Leishmania* and determine their prevalence in specimens collected near to the Chiapas-Guatemala border. Obtaining information on the microbiome of this phlebotomine fauna possibly helps to evaluate its possible use as a biological control method for sand flies, helping to prevent the transmission of leishmaniasis.

## Material and method

### Study area and specimens analyzed

**Fig. 1:**
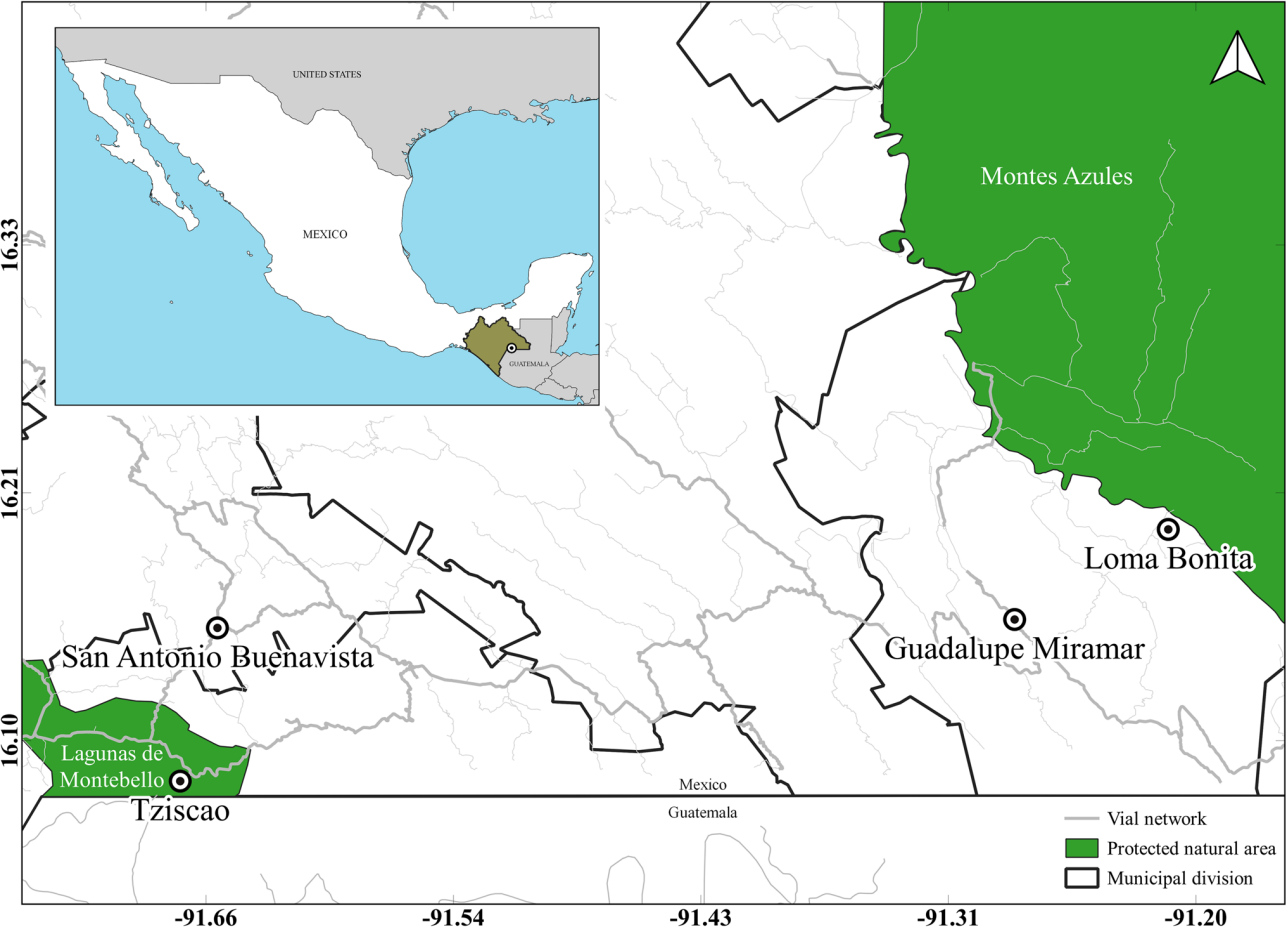
Map of the geographic location of the sampled areas in Chiapas, Mexico

We analyzed sand fly specimens that had previously been collected in areas with an elevated risk of transmission of leishmaniasis in the state of Chiapas, near the border between Mexico and Guatemala, during the period 2009–2011. The localities that were analyzed included (1) San Antonio Buenavista (16.1523 N; −91.6497 W), (2) Tziscao (16. 0812 N; −91. 6670 W), (3) Guadalupe Miramar (16. 1562 N; −91.2792 W), and (4) Loma Bonita (16. 1980 N; -91.2078 W) (Fig. 1).

All analyzed specimens were collected using CDC light traps (Mod. 512) and modified Magoon traps baited with bite-protected humans. The traps were located in five houses (indoors and peridomicile) in four transects ranging 500 m from the edge of the houses into the surrounding vegetation. During the winter months (October–March) traps were used between 18:00 and 06:00 h and in the summer months (April–September), the traps were active between 19:00 and 07:00 h (Ibáñez-Bernal et al. 2015). The collected sand flies were stored in 70% ethanol, the head was dissected for morphological identification, and the remaining parts of the body were used for molecular analysis. Permanent mounting was done following published protocols (Ibáñez-Bernal 2005), and the identification and classification of specimens were based on Galati (2019) proposal. We use the abbreviation system proposed by Marcondes (2007).

### DNA extraction and polymerase chain reaction (PCR) conditions

DNA was only extracted from the females, using a plasmid extraction protocol modified by Pech-May et al. (2013). Since only small amounts of DNA were available, we first analyzed sand fly DNA pools of 10 specimens of the same species and the same localities. If these tested positive, the specimens of the pools were individually analyzed. For molecular detection of *Wolbachia* strains, we amplified a fragment of the surface protein (*wsp*) gene of ~ 600 bp using the primers *wsp* 81F (5′ TGG TCC AAT AAG TGA TGA AGA AAC 3′) and *wsp* 691R (5′ AAA AAT TAA ACG CTA CTC CA 3′) (Braig et al. 1998). The PCR was performed with an initial denaturation at 95 °C for 5 min, followed by 35 cycles at 94 °C for 1 min, 55 °C for 1 min, and 72 °C for 1 min; with a final extension at 72 °C for 5 min. For the detection of *Bartonella* species, we amplified a segment of ~ 378 bp of the citrate synthase (*glt*A) gene, using the primers BhCS871.p (5′-GGG GAC CAG CTC ATG GTG G -3′) and BhCS1137.n (5′-AAT GCA AAA AGA ACA GTA AAC A-3′) (Norman et al. 1995). The PCR was performed under the following conditions: initial denaturation at 95 °C for 5 min, followed by 35 cycles at 95 °C for 30 s, 51 °C for 30 s, 72 °C for 30 s, and a final extension at 72 °C for 7 min (Rubio et al. 2014). Additionally, for the analysis of a possible coinfection of *Leishmania* with the bacteria, we amplified a ~ 350 bp fragment of the gene ITS1 for the detection of *Leishmania*, using the primers LITSR (5′-CTG GAT CAT TTT CCG ATG—3′) and L5.8S (5′-TGA TAC CAC TTA TCG CAC TT—3′), using previously reported PCR conditions (El Tai et al. 2001; Lozano-Sardaneta et al. 2020).

The reaction mixture was prepared in a final volume of 25 μl containing 12.5 μl GoTaq® Green Master Mix 2X Promega Corporation (Madison, WI, USA), 1 μl of each primer (100 ng each), 5 μl DNA template (~50 ng/μl), and 5.5 μl nuclease-free water. The negative control consisted of ultrapure water instead of DNA. The PCR reactions were performed in a Veriti 96 Well Thermal Cycler (Applied Biosystems^TM^, Thermo Fisher Scientific, USA). The amplified products were analyzed by electrophoresis in 2% agarose gels stained with 0.4µL of Midori Green Advance (Nippon genetics). The positive PCR products were purified and sequenced at Laboratorio de Secuenciación Genómica de la Biodiversidad y de la Salud, Instituto de Biología, UNAM.

### Data analysis

The *gltA* and *wsp* electropherograms were visualized and edited in the software Chromas. Each sequence was compared with all the sequences available at the NCBI database, using BLASTn (https://blast.ncbi.nlm.nih.gov/Blast.cgi) as a preliminary confirmation.

The retrieved sequences were aligned with other reference sequences deposited on GenBank using MEGA version X (Kumar et al. 2018). For the phylogenetic analysis of the sequences of both genes, we used a maximum likelihood (ML) reconstruction performed in MEGA X, with 10,000 bootstraps, using the Tamura 3 parameters (T92)+ Gamma distribution substitution model, showing a BIC score of 5117.294 (for *glt*A in *Bartonella*) and BIC 8424.828 (for *wsp* of *Wolbachia*). The genetic distances were calculated in MEGA X. For the *wsp* gene, we translated the sequences into amino acids to facilitate the alignment (Zhou et al. 1998). The obtained sequences were deposited in GenBank under the following accession numbers *Wolbachia wsp* OP618079-OP618084 and *Bartonella gltA* OP618073-OP618079.

## Results

### Sand fly specimens analyzed

A total of 531 sand fly specimens were recovered (Table 1), belonging to six genera and 10 species. The most abundant species were *Psychodopygus panamensis*, *Pintomyia ovallesi*, and *Dampfomyia deleoni*. Guadalupe Miramar was the locality with the highest numbers of specimens available for molecular analysis.

**Table 1:** List of analyzed species of female sand flies collected in four localities of Chiapas, Mexico

Species	Loma Bonita	Guadalupe Miramar	San Antonio Buenavista	Tziscao	Total
*Ps. bispinosa* (Fairchild & Hertig)	0 (0/0)	2 (0/0)	1 (0/0)	0 (0/0)	3
*Pa. carpenteri* (Fairchild & Hertig)	0 (0/0)	2 (0/0)	0 (0/0)	0 (0/0)	2
*Ps. corossoniensis* (Le Pont & Pajot)	0 (0/0)	7 (0/3)	0 (0/0)	0 (0/0)	7
*Lu. cruciata* (Coquillett)	13 (0/4)	7 (0/0)	4 (0/0)	1 (0/0)	25
*Da. deleoni* (Fairchild & Hertig)	27 (0/0)	25 (0/0)	0 (0/0)	0 (0/0)	52
*Da. delpozoi* (Vargas & Díaz-Nájera)	0 (0/0)	2 (0/0)	0 (0/0)	0 (0/0)	2
*Pi. ovallesi* (Ortiz)	106 (27/2)	1 (0/0)	1 (0/0)	0 (0/0)	108
*Ps. panamensis* (Shannon)	42 (0/0)	244 (0/24)	0 (0/0)	0 (0/0)	286
*Pa. shannoni* (Dyar)	4 (0/0)	0 (0/0)	3 (0/0)	0 (0/0)	7
*Ny. ylephiletor* (Fairchild & Hertig)	8 (0/2)	31 (0/11)	0 (0/0)	0 (0/0)	39
Total	200	321	9	1	531

*Ps:* *Psychodopygus*, *Pa:* *Psathyromyia*, *Lu:* *Lutzomyia*, *Da:* *Dampfomyia*, *Pi:* *Pintomyia*, *Ny:* *Nyssomyia*

In each locality, we indicate the number of insects (number positive for *Wolbachia*/number positive for *Bartonella*)

### *Wolbachia* detection

*Wolbachia* strains that were detected in the sand flies showed a prevalence of 8.6% (46/531). *Wolbachia* was detected in *Ps. corossoniensis*, *Lu. cruciata*, *Pi. ovallesi*, *Ps. panamensis*, and *Ny. ylephiletor* from Guadalupe Miramar and Loma Bonita. The prevalences were *Ps. corossoniensis* (3/7; 42.8%), *Lu. cruciata* (4/25; 16%), *Pi. ovallesi* (2/108; 1.85%), *Ps. panamensis* (24/286; 8.4%), and *Ny. ylephiletor* (13/39; 33.33%). The highest number of infected specimens was found in Guadalupe Miramar (32/46).

The sequences showed similarities with other strains previously reported in sand flies. Thus, *Wolbachia* detected in *Lu. cruciata* was 99.81% similar to *Wolbachia* endosymbiont of *Pa. shannoni* (MT533592.1), and the *Wolbachia* detected in *Ps. panamensis* was 100% identical to *Wolbachia* endosymbiont of *Micropygomyia stewarti* (KJ174699.1).

Additionally, we observed similarities with other strains detected in mosquitoes of the genus *Culex* sp. and the parasitoid wasp. Thus, the *Wolbachia* detected in *Ny. ylephiletor* was 99% similar to *Wolbachia* of *Belonocnema treatea* (wasp) (MG252474.1), and the *Wolbachia* strain detected in *Ps. corosoniensis* and *Ps. panamensis* was 100% similar to *Wolbachia* endosymbiont of *Culex quinquefasciatus* (LC276757.1) and *Culex pipiens* (KT964225.1), respectively.

**Fig. 2:**
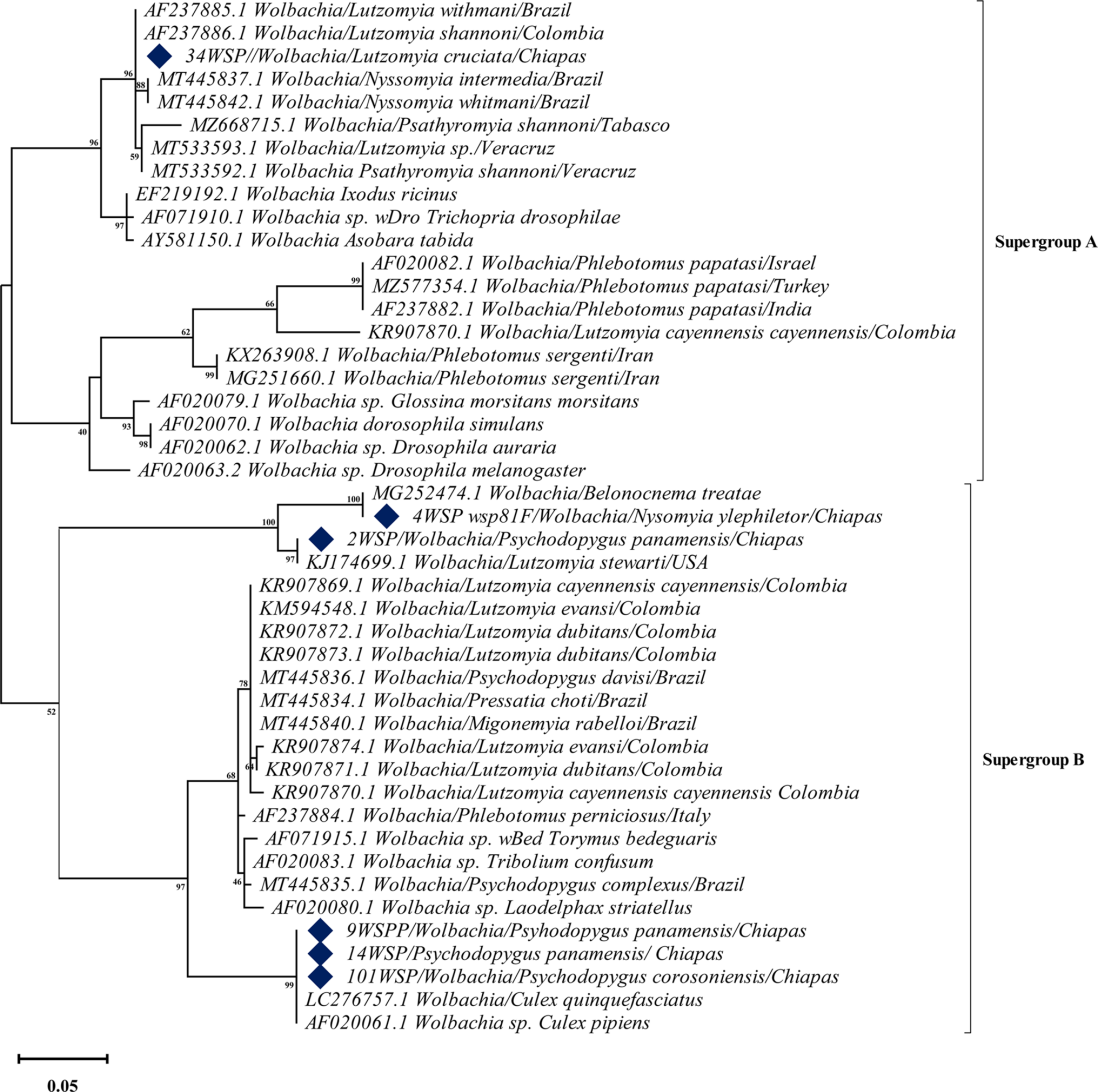
Phylogenetic analysis using maximum likelihood for the *Wolbachia wsp* gene in sand fly specimens collected in Chiapas*.* The obtained sequences are marked with a diamond

According to the ML analysis (Fig. 2), we detected one *Wolbachia* strain belonging to the supergroup A. This *Wolbachia* strain *w*Whi was detected in *Lu. cruciata* species (collected in Loma Bonita). It had previously been recorded in *Ny. intermedia*, *Pa. shannoni*, and *Ny. whitmani* from Brazil, Mexico, and Colombia, showing a bootstrap value of 98%, and a genetic distance ranging from 0 to 2.5%. Although an earlier study had reported *Wolbachia* in *Lu*. *cruciata* from Chiapas (Mikery-Pacheco et al. 2012), the molecular identity of this strain had not been shown.

We also detected three strains belonging to the supergroup B: (1) the *w*Tre4 strain detected in *Ny. ylephiletor* (collected in Guadalupe Miramar). It had previously been reported in the parasitoid wasp of oak trees *Belonocnema treatea*, showing a bootstrap value of 100%; (2) the *w*Ste strain detected in *Ps. panamensis* (collected in Guadalupe Miramar). It had previously been reported in *Micropygomyia stewarti*, showing a bootstrap value of 97%; and (3) the strain *w*Pip detected in *Ps. panamensis* (collected in Loma Bonita and Guadalupe Miramar) and in *Ps. corossoniensis* (collected in Guadalupe Miramar) showing a bootstrap value of 99%. This species had also been reported in mosquitoes of the genus *Culex* from India (LC276757.1) and Tunisia (AF020061.1) (Fig. 2).

### *Bartonella* detection

**Fig. 3:**
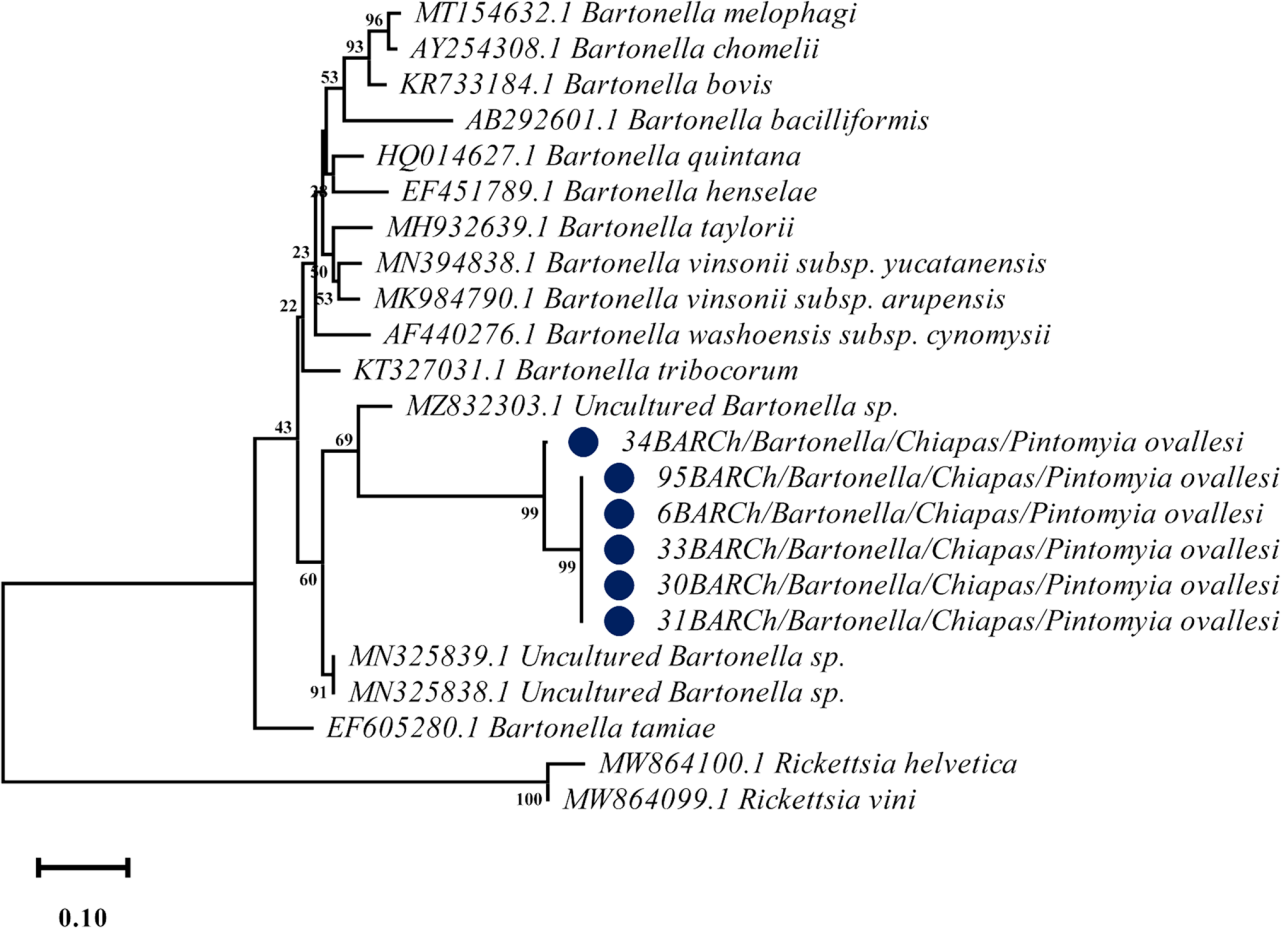
Phylogenetic analysis of the *Bartonella glt*A gene detected in sand fly specimens, collected in Chiapas, using maximum likelihood*.* The obtained sequences are marked with a circle

The bacterium *Bartonella* sp. was detected in 27/531 specimens, showing a prevalence of 5.08%. The only positive species was the sand fly *Pi. ovallesi* from Loma Bonita, Chiapas. The sequences were 98% similar to each other and showed 83% similarity with a *Bartonella* sp. of *Lutzomyia* sp. from Mexico (MN325839.1). The ML analysis (Fig. 3) showed that the sequences correspond to a new lineage of *Bartonella* sp. associated with *Pi. ovallesi*, showing a bootstrap support value of 99% and genetic distances ranging 0 to 3.4%. It clustered in a clade with another lineage that had previously been recorded in sand flies of Mexico, showing a bootstrap value of 60% and genetic distances ranging from 25 to 29% with regard to other *Uncultured Bartonella* sp. from the states of Veracruz and Tamaulipas. This clade seems to be separated from the other pathogenic *Bartonella* species (Fig. 3). Since there are only few records on *Bartonella* species associated with sand flies outside of endemic areas, more studies are necessary to delimit and characterize these bacterial species associated with phlebotomine sand flies.

Although we analyzed the sand flies for infections with *Leishmania* spp., none of the specimens tested positive. Thus, our study ruled out the coinfection of these bacteria and *Leishmania* in sand flies of Chiapas.

## Discussion

Phlebotomine sand flies have a relevant role in vector-borne diseases worldwide. Gaining insights into their microbiome helps reveal biological aspects of their cycle including reproduction, immune system, vectorial capacity, fitness, survival, and competence (Vivero et al. 2019). The gut microbiota of sand flies is involved in a wide range of biological and physiological processes, which could hamper or facilitate pathogen transmission, since some bacteria can negatively affect pathogen colonization (Louradour et al. 2017). The microbiome is closely related to the nutrition of sand flies, which can acquire the microorganisms from the soil, plants, and blood during their development and feeding. Variations of the intestinal microbiota can play a role in the survival and colonization of some parasites in the gut of sand flies (Louradour et al. 2017; Vivero et al. 2019).

In Mexico, studies on microorganisms associated with sand flies are scarce. Yet, *Wolbachia* and *Bartonella* had previously been reported in sand flies (Mikery-Pacheco et al. 2012; Lozano-Sardaneta et al. 2019, 2021a, b; Lozano-Sardaneta et al. 2021a, 2022). This led us to retrospectively analyze the possible presence of bacteria in sand flies species that had previously been collected in Chiapas, Mexico, a state with a high prevalence of leishmaniasis.

We now detected four *Wolbachia* strains (*w*Whi, *w*Tre4, *w*Pip, and *w*Ste) in five sand fly species from Chiapas. These *Wolbachia* strains had previously been reported in other sand fly species and other insects, showing a prevalence of 8.6%. This is higher compared to other studies carried out in Mexico, where a prevalence of 0.98% has been reported (Mikery-Pacheco et al. 2012; Lozano-Sardaneta et al. 2021a, b; Lozano-Sardaneta et al. 2022). Generally, *Wolbachia* strains are widely distributed in wild populations of arthropods, causing reproductive alterations to ensure their own propagation by vertical transmission, thereby infecting a high number of specimens (Ono et al. 2001).

The ML analysis showed that the *Wolbachia* strain *w*Pip was detected in *Ps. panamensis* in two distant localities (Guadalupe Miramar and Loma Bonita) and in *Ps. corossoniensis*. This strain has also been reported in species of mosquitoes of the genus *Culex*, where it provides protection against viral infections (Fraser et al. 2020). Additionally, we detected the *Wolbachia w*Ste strain in *Ps. panamensis*. This *Wolbachia* strain had previously been reported in the sand fly *Mi. stewarti* in the USA and shown to confer protection to the sand fly against *Plasmodium* (Hughes et al. 2014). The role of these strains in species of the genus *Psychodopygus* is unknown, yet is likely that they could prevent the establishment of some viruses. Although the *Wolbachia w*Whi strain has previously been reported in *Nyssomyia intermedia*, *Nyssomyia whitmani*, and *Pa. shannoni* and from Brazil, Colombia, and Mexico (Ono et al. 2001; Lozano-Sardaneta et al. 2022), this is the first time that this strain is recorded in *Lu. cruciata*. Interestingly, in this study, this strain was not found in *Pa*. *shannoni*, a sand fly where it has previously been reported in Mexico (Lozano-Sardaneta et al. 2021a, b; Lozano-Sardaneta et al. 2022). The transmission of the *Wolbachia w*Whi strain is generally occurs by horizontal transmission between closely related species, which excludes the sand fly *Lu. cruciata*, since it belongs to another genus (Ono et al. 2001; Karimian et al. 2018).

The *Wolbachia* strain *w*Tre was detected in the sand fly *Ny. ylephyletor*. This *Wolbachia* strain has also been associated with the parasitoid wasp *B*. *treatea* in the USA and could have been acquired by horizontal transmissions from the oak tree (Fagaceae: *Quercus*) or other parasitoids, causing reproductive isolation in their host (Schuler et al. 2018).

Although the vertical transmission of *Wolbachia* strains is common, in some cases a horizontal transmission also occurs, such as across parasitoids, invertebrate predators, ectoparasitic mites, and host plants or food sources (Vavre et al. 1999; Li et al. 2017). In the case of sand flies, the plant-mediated horizontal transmission in sand fly species seems a plausible potential transmission pathway. It has been confirmed that when different insect taxa feed on the same plant and share resting sites, they can acquire the same endosymbionts (Sintupachee et al. 2006; Li et al. 2017). Since phlebotomine sand fly males and females feed on sugar from plants (nectar and/or phloem sap), as well as on sugars excreted by “honeydew” aphids (Lima et al. 2016; Abbasi et al. 2018), this could explain why the same *Wolbachia* strains show widespread distributed in other taxa, and in phlebotomine sand flies from different localities from Chiapas, Mexico. It is therefore important to take the horizontal transmission route into account, since it could introduce new phenotypes in the different hosts. This could enable different fitness benefits, such as increasing the resistance against particular pathogens or inducing speciation, depending on the sand fly species (Monteiro et al. 2016; Li et al. 2017; Abbasi et al. 2018; Schuler et al. 2018). Therefore, complementary studies are necessary to test the relevance of horizontal transmission of *Wolbachia* strains in sand flies.

We now also detected, for the first time, a new lineage of *Bartonella* sp. associated to *Pi. ovallesi* in Loma Bonita, Chiapas. The sand fly *Pi. ovallesi* is anthropophilic and considered to be a vector for *Leishmania* spp. in Belize, Colombia, Panama, and Venezuela. In Mexico, this sand fly was recently found to be infected with *Leishmania* and is now regarded as a potential vector for the parasite (Lozano-Sardaneta et al. 2022). According to the ML analysis, the sequences obtained for the *Bartonella* sp. correspond to a new lineage that grouped into a clade with other Uncultured *Bartonella* spp. associated with sand flies in Mexico (Lozano-Sardaneta et al. 2019; Lozano-Sardaneta et al. 2021b). Even though the ML analysis showed a genetic difference of 29%, this lineage is included in the genus *Bartonella*. A *Bartonella* species can be classified as new, if it exhibits less than ≥ 96.0% nucleotide similarity using the *glt*A gene with other validated species (La Scola et al. 2003). In general, *Bartonella* species have host specificity, which suggests that specific adaptations are involved in the successful establishment and survival in a new arthropod vector or mammal host (Chomel et al. 2009). It is probable that the new *Bartonella* lineage detected in our study is specific for *Pi. ovallesi* and could have been acquired during the blood feeding.

The fact that we did not observe the presence of *Leishmania* in any of the analyzed sand fly species is consistent with other records of Mexico. None of the studies has shown the coinfection of *Bartonella* with *Leishmania* in sand flies (Lozano-Sardaneta et al. 2021b). Undoubtedly, more studies are needed to determine whether infection of sand flies with this bacterium possibly avoids the establishment of *Leishmania* in the same sand fly.

Although the detection of bacteria is relevant for the study of phlebotomine sand flies due to their potential use as biological controls to prevent the transmission of *Leishmania*, it is difficult to characterize all the species. We now report for the first time the presence of four *Wolbachia* strains associated to sand flies from Chiapas, Mexico, showing a high prevalence. It is probable that *Wolbachia* may confer some benefits when it is transmitted horizontally, possibly by decreasing the transmission of parasites and viruses. Furthermore, we now report a new lineage of *Bartonella* in sand flies, although we cannot confirm if the sand flies are vectors of this bacterium, since to date all infected sand fly species have shown to harbor different *Bartonella* lineage. Clearly, further studies are required to elucidate the possible role of sand fly species as potential vectors of other *Bartonella* species, to help understand their transmission pathway, and to improve their molecular identification.

## Data Availability

The sequences generated and analyzed in this study have been deposited in the GenBank database under the accession numbers: OP618073-OP618084.
